# *Rhizobium pongamiae* sp. nov. from Root Nodules of *Pongamia pinnata*

**DOI:** 10.1155/2013/165198

**Published:** 2013-07-02

**Authors:** Vigya Kesari, Aadi Moolam Ramesh, Latha Rangan

**Affiliations:** Department of Biotechnology, Indian Institute of Technology Guwahati, Assam 781 039, India

## Abstract

*Pongamia pinnata* has an added advantage of N2-fixing ability and tolerance to stress conditions as compared with other biodiesel crops. It harbours “rhizobia” as an endophytic bacterial community on its root nodules. A gram-negative, nonmotile, fast-growing, rod-shaped, bacterial strain VKLR-01^T^ was isolated from root nodules of *Pongamia* that grew optimal at 28°C, pH 7.0 in presence of 2% NaCl. Isolate VKLR-01 exhibits higher tolerance to the prevailing adverse conditions, for example, salt stress, elevated temperatures and alkalinity. Strain VKLR-01^T^ has the major cellular fatty acid as C_18:1_ **ω**7c (65.92%). Strain VKLR-01^T^ was found to be a nitrogen fixer using the acetylene reduction assay and PCR detection of a *nif*H gene. On the basis of phenotypic, phylogenetic distinctiveness and molecular data (16S rRNA, *rec*A, and *atp*D gene sequences, G + C content, DNA-DNA hybridization etc.), strain VKLR-01^T^ = (MTCC 10513^T^ = MSCL 1015^T^) is considered to represent a novel species of the genus *Rhizobium* for which the name *Rhizobium pongamiae* sp. nov. is proposed. *Rhizobium pongamiae* may possess specific traits that can be transferred to other rhizobia through biotechnological tools and can be directly used as inoculants for reclamation of wasteland; hence, they are very important from both economic and environmental prospects.

## 1. Introduction

*Pongamia pinnata* (L.) Pierre is a nonedible “pioneer” biodiesel and medicinal tree species of the family Leguminosae that grows in multiple geoclimatic conditions, ranging from humid, tropical, subtropical regions to cooler and semiarid zones [1–3]. Nitrogen is an important nutrient for plant growth and yield; however, its availability in soils is limited. Modern agriculture depends on chemically synthesized N fertilizers which are expensive and require fossil fuels for production, adding to greenhouse gas emissions. Biological nitrogen fixation is a useful and important alternative [4], especially in biofuel production [2, 5]. *Pongamia* can grow on low-fertility land due to its nodulation properties and good N_2_-fixing symbiotic associations with “rhizobia” (a polyphyletic assemblage of alphaproteobacteria family: *Rhizobiaceae*), thus minimizing competition with food crops or related fertilizer, water, and land resources needed for food and fodder production [6]. The sustainable production of plant oils for biodiesel production from a tree crop such as *P. pinnata*, which can be cultivated on marginal lands, has the potential to not only provide a renewable energy resource but in addition alleviate the competitive situation that exists with food crops as biofuels and associated arable land and water use. It is also used in agriculture and environmental management, due to its insecticidal and nematicidal properties [7]. Finally, *Pongamia* has been identified as a resource for agroforestry, urban landscaping to suspend the pollutants and the bioameloriation of degraded lands. 

Isolation and identification of authentic and effective rhizobia isolates are required to support *P. pinnata* plantations in nitrogen-poor soils. *Pongamia* trees are purportedly able to grow in a wide range of environments: in the tropics, with temperatures from 13–45°C, saline soils, and in soils with a range of pH including sodic soils, and they are an ideal candidate for reforestation of marginal lands [3, 5]. The ability of rhizobia to grow in these diverse environmental conditions will be important for the establishment and success of *Pongamia* plantations on these unfertile lands. It, thus, satisfies all “sustainability criteria” expected from modern second- and third-generation biofuel crops. However, little attention has been paid to the occurrence of nitrogen-fixing endophytic bacteria in the rhizospheres of this biodiesel tree which is important for their diverse applicabilities as well as agronomic and ecological significances. In preliminary studies, the effective nodulations of *P. pinnata* with three strains of rhizobia (*Bradyrhizobium japonicum* strain CB1809, *Bradyrhizobium sp.* strain CB564, and *Rhizobia sp.* strain NGR234) were demonstrated [1].

The importance of characterizing indigenous rhizobia of *Pongamia* cannot be overemphasized. Still there has not been any detailed study of phenotypic characteristics and symbiotic effectiveness of rhizobia isolates which naturally nodulate *Pongamia* considering its potential value in sustainable agriculture and role in agroforestry. Therefore, in this work, we attempt to isolate the nitrogen-fixing rhizobial symbiont strain from nodules of *P. pinnata* occurring in North Guwahati, Assam, India. The objectives were to determine the exact taxonomic position of isolated and identified strain by using a polyphasic characterization that included determination of phenotypic and biochemical properties, phylogenetic investigations based on 16S rRNA, *atp*D, and *rec*A gene sequences, and genetic analysis. Further investigations were also performed in order to verify the nodulation and nitrogen-fixing property of the isolated bacterium strain. 

## 2. Materials and Methods

### 2.1. Soil Sampling and Isolation of Rhizobia

*P. pinnata* saplings approximately 2-3 months old found in North Guwahati (26°14′6′′ N; 91°41′28′′ E), Assam, India, were uprooted during April 2010 containing distinct nodules. Nodules excised from the roots were surface sterilized with 70% (v/v) ethanol for 1 min. Subsequently, nodules were treated with 10% (w/v) sodium hypochlorite for 15 min and washed with sterile distilled water (3x). Single surface-sterilized nodule (approximately 2 mm) was opened into two halves with a sterile blade, and the central parts of the nodule were scooped with blunt needle, macerated, and diluted in 500 *μ*L of saline water (0.9%). Roughly 100 *μ*L of the inoculum was spread on yeast extract-mannitol (YEM) and tryptone-yeast extract (TYE) agar plates and incubated at 28°C for 1–3 days. The purity of the culture was verified by repeated streaking of single colony onto YEM agar [8] with 25 mg Kg^−1^ (w/v) congo red. Single purified isolate was maintained in YEM broth containing 20% (v/v) glycerol at −80°C.

### 2.2. Growth and Phenotypic Characteristics

Cell size and morphology of the root nodule isolate were determined using scanning electron microscopy (LEO 1430 VP; Leo Electron Microscopy, Ltd., Cambridge, the UK) at 10 kV. For the micromorphology study, cells from the exponential growth phase (grown in YEM broth at 28°C) were harvested by centrifugation and fixed in 2.5% (w/v) glutaraldehyde for 45 min. The cells were washed with phosphate-buffered saline (PBS) and applied to ethanol dehydration series (at 50%, 70%, 90%, and 100% for 10 min each) (v/v) followed by critical-point drying with CO_2_ and sputter-coating with gold as described by Boyde and Wood [15]. A growth characteristic of isolate was recorded at different temperatures (4, 25, 28, 30, 37, 42, and 50°C) in YEM broth and agar until 48 h of culture. The ability to grow in acid and alkaline media was also tested by inoculating the isolate onto YEM broth and YEM agar plates adjusted to various pH values (pH 4.0–11.0 at intervals of 1 pH units) using 1 N HCl/1 N NaOH. The NaCl tolerance of the isolate was tested by growing in YEM broth and YEM agar plates containing 0%, 1%, 2%, 3%, 4%, and 5% (w/v) NaCl. 

### 2.3. Gram Staining: Biochemical and Physiological Characteristic

Gram reaction was determined with the bioMérieux Gram-stain kit according to the manufacturer instructions. Acid production from different carbohydrates was determined by employing the API 50 CH system (bioMérieux) according to the manufacturers instructions. The ability of the isolate to utilize various carbon and amino acids compounds as sole carbon and nitrogen source was investigated using the method described by Lindström and Lehtomäki [16]. Def 9 (carbon source) and Def 8 (nitrogen source) agar mediums were used, and appropriate controls were maintained. Results were noted after 3 days of incubation at 28°C. A set of physiological characteristics including catalase and oxidase tests and nitrate reduction were assessed using protocols described by Shieh et al. [17]. Gelatin hydrolysis and methyl red test were also performed using the methods of Smibert and Krieg [18]. The intrinsic antibiotic resistance tests for the isolate were performed by disc-diffusion assay in YEM agar against 16 different antibiotics (Discs, HiMedia) of concentrations ranging from 2 to 100 *μ*g. Cell biomass for the analysis of isoprenoid quinones was obtained from the isolate grown on yeast extract-mannitol broth (YEM) (12 h, 28°C, 180 rpm).

### 2.4. FAME Analysis

Fatty acid methyl esters (FAMEs) of the isolate were extracted and prepared according to the standard protocol of the MIDI Sherlock/Hewlett Packard Microbial Identification System as described by Sasser [19]. For cellular FAME analysis, isolate was grown on trypticase soy broth agar (TSBA), which consists of 30 g trypticase soy broth and 15 g of agar (BBL) for 12 h at 28°C under aerobic conditions (180 rpm). The fatty acid methyl esters extracts were analyzed by Gas Chromatography MODEL 6850 (Agilent Gas Chromatography) equipped with an Agilent ultra 2 capillary column. 

### 2.5. DNA Extraction, PCR Amplification, and Sequencing

Cell biomass for DNA extraction was obtained from the isolate grown on yeast extract-mannitol (YEM) broth (12 h, 28°C, 180 rpm). Chromosomal DNA was isolated and purified using Sigma's GenElute Bacterial Genomic Kit. The 16S rRNA gene was amplified by PCR using two consensus primer fD1 and rD1 [20]. Polymerase chain reaction (PCR) of 16S rRNA gene was performed in 25 *μ*L volume mixing the template DNA (10 ng) with 1x PCR buffer (Bioline), 2.5 mM MgCl_2_ (Bioline), 0.5 U *Taq* DNA polymerase (Bioline), 2.5 mM dNTPs each, 0.4 *μ*M (each) primers fDl and rDl using a DNA thermal cycler (Applied Biosystems, USA). The following temperature profile was used for DNA amplification: an initial denaturation at 95°C for 5 min followed by 35 cycles of 94°C for 1 min, 55°C for 1 min, and 72°C for 2 min and a final extension step of 72°C for 10 min. Reaction products were electrophoresed on a 1.3% (w/v) agarose gel and were purified using a Qiaquick PCR purification kit (Qiagen) before sequencing. Sequencing reactions were performed using the ABI PRISM Dye Terminator Cycle Sequencing Kit (Applied Biosystems, USA) with the primers IRF1, 1050R, 800F, and 800R [21], and they were analyzed in an automatic sequencer ABI PRISM 3730 sequencer (Applied Biosystems). PCR amplifications of other housekeeping genes *rec*A and *atp*D were performed under the conditions described by Yoon et al. [9]. The primer sequences that were used for amplification and sequencing of 16S rRNA, *rec*A, and *atp*D genes are listed in (Supplementary Table 1; see Supplementary Material available online at http://dx.doi.org/10.1155/2013/165198.) The sequences of these genes were compared with the sequences available from GenBank using BLASTN program [22] and were aligned using ClustalW2 multiple sequence alignment [23]. Phylogenetic trees were inferred using the neighbor-joining method [24], and distances were determined according to the Kimura-2 model [25]. Bootstrap analysis was based on 1000 resamplings. The MEGA 4.0 version [26] was used for all analyses. 

### 2.6. Nitrogen Fixation and Nodulation Assessment Test

The acetylene reduction assay (ARA) was used to test the isolate for potential nitrogen fixations. The amount of ethylene produced was measured using 10% (v/v) acetylene according to the method of Li and MacRae [27] using a Hewlett Packard 4890 GC equipped with a Porapak N column. Isolate was subjected to *nif*H-specific PCR amplification using the primers (Supplementary Table 1) of Poly et al. [28]. Nodulation test for isolate was performed by PCR amplification of *nod*D gene (Supplementary Table 1) as described by Yoon et al. [9].

### 2.7. DNA Hybridization and G + C Content

The DNA G + C content was determined as described by Tamaoka and Komagata [29]. Isolate was disrupted using a French pressure cell (ThermoSpectronic), and the DNA in the crude lysate was purified by chromatography on hydroxyapatite as described by Cashion et al. [30]. DNA was hydrolyzed, dephosphorylated, and analyzed for its G + C content by HPLC [31]. Nonmethylated Lambda DNA (Sigma) was used as a reference. DNA-DNA hybridization was assessed for isolate against reference strain *Rhizobium radiobacter* DSM 30147^T^ (= AB247615) that showed 97% sequence similarity. DNA-DNA hybridization was carried out as described by De Ley et al. [32] under consideration of the modifications described by Huss et al. [33] using a model Cary 100 Bio UV/VIS spectrophotometer equipped with Peltier-thermostatted 6 × 6 multicell changer and a temperature controller with *in situ* temperature probe (Varian). The highest and lowest values obtained were excluded, and the means of the duplicates were quoted as DNA-DNA relatedness values. Analysis of respiratory quinones was carried out by the Identification Service and Dr. Brian Tindall, DSMZ, Braunschweig, Germany, according to the method of Komagata and Suzuki [34].

## 3. Results and Discussion

Since *P. pinnata* is introduced as the most important multipurpose tree for biodiesel production, it has become the most widespread legume in India and other parts of the world. This predominance has resulted from the massive implantation of the species for multipurpose use in a broad edaphic range including urban and social forestries to alleviate the environmental imbalance. The community structure of *Pongamia* root-nodule bacteria has been addressed by a few studies that assessed nodulation ability by endogenous rhizobia and also few strains commonly associated with *Glycine max* and their stimulatory effect on nodule number and plant growth [1, 35]. In this present study, we extend the work on *Pongamia* root-nodulating bacteria to isolate and characterize the novel *Rhizobium* species with traits that make it competitive in stress environments from the root nodules of biodiesel crop *P. pinnata* from North Guwahati, Assam, India. 

### 3.1. Trapping and Isolating Root-Nodulating Bacteria

Root nodules were observed in all of the *Pongamia* saplings uprooted from the sampling site, indicating that root nodules occur widely in this legume crop growing naturally in North Guwahati, Assam, India. The plants, however, varied in the extent to which they were nodulated as nodules varied in shapes and sizes formed on primary as well as secondary roots (Supplementary Figure 1). Different shapes of root nodules of *P. pinnata* observed in the present study may be related to different developmental phases of the nodule ontogeny, and it is, therefore, not surprising that the nodule morphology has been used as a taxonomic marker [36]. Phenotypic traits of *Pongamia* saplings, namely, mean shoot length, mean root length, and the number of nodules observed in *P. pinnata,* were 28.25 ± 3.24, 12.17 ± 2.19, and 9.90 ± 2.31, respectively. Transverse section of single nodule (approximately 20 *μ*m thick) in SEM image revealed the absence of any visible bacteria in the outer wall portion of the nodule. But when the middle portion was focused, each cell was fully filled with rod-shaped bacteria (Supplementary Figures 2A and 2B). Therefore, the middle portion of the root nodule was further used for isolation of single pure bacterial colony specific to *P. pinnata* and named as VKLR-01.

### 3.2. Growth, Phenotypic, Biochemical and Physiological Characteristics of Isolate

The first visible growth of the bacterium was observed as a small white shiny dot-like structure which increased in size from 1.5–3.5 mm (24 h) to 4.0–5.5 mm (48 h) in both YEM and TYE plates at 28°C (Supplementary Figure 3A). The generation time noted was 0.67 h in YEM medium. Pure culture was obtained from individual colony designated as VKLR-01. Isolate VKLR-01 showed creamy or white opaque, round or convex, and gummy colonies, with little or moderate extracellular polysaccharide production (EPS) having a diameter of 1.5–3.5 mm after growth for 24 h at 28°C. Metabolism is strictly aerobic. Isolate VKLR-01 appears to be a fast-growing rhizobial strain, forming colonies of 4–5.5 mm in diameter in 2-day time. Existing reports show that trees are as often nodulated by fast-growing as by slow-growing rhizobia [37]. 

The SEM image of the purified isolate VKLR-01 from an exponential phase revealed the bacterium to be rod shaped, nonmotile having a cell dimension of 0.4-0.5 *μ*m width and 1.4–1.6 *μ*m length, respectively (Supplementary Figure 3B). Isolate VKLR-01 occurs at temperature range of 25–30°C (optimal at 28°C) and can tolerate up to 42°C, but no growth at 4 and 50°C. Isolate VKLR-01 can tolerate the salt concentration varied in the range of 1%–4% NaCl, but no visible growth was observed at 5% NaCl concentration in the YEM medium. The isolate also grew at pH 6.0 to pH 11.0, but no visible growth was observed at and below pH 5.0. Optimum growth conditions for the isolate VKLR-01 were temperature of 28–30°C, pH of 7.0–8.0, and 2% (w/v) NaCl. Temperature is known to influence survival, growth, and nitrogen fixation of *Rhizobium* [38]. Isolate VKLR-01 can tolerate extreme environmental conditions such as temperature up to 42°C and 4% NaCl, which differentiates this strain from other species. Similar results were found with rhizobia that nodulate *Lotus corniculatus* [39]. Generally, rhizobia collected from high temperature areas are resistant to high temperatures, and their tolerance is probably due to their adaptation to the extreme air temperatures inherent of tropical climate [40]. Salinity tolerance of the host is often the limiting factor in determining effective symbiosis of compatible rhizobia under saline conditions [41]. As the isolate VKLR-01 is able to adapt even in the presence of such unfavorable environmental conditions, this strain may be used for generation of genetically modified rhizobia by using genetic engineering tools. There is a report available that showed transferring of a 10 kb DNA fragment constructed from a wild-type strain of *Sinorhizobium *to *Rhizobium etli* (a sensitive strain) having resistance to several antibiotics, 4% NaCl, low and high pHs, heavy metals, and a temperature as high as 43°C [42]. Another application of this strain would be as an inoculum for a number of crop legumes that will significantly improve nodulation and nitrogen fixation and may lead to increase in plant dry matter under a low-level N fertilizer in low-fertility land. 

Isolate VKLR-01 is Gram negative and nonspore forming. Gas is not produced from raffinose, sucrose, arabinose, mannitol, lactose, and glucose. Acid was produced from fermentation of sucrose, mannitol, and lactose but not from raffinose, arabinose, and glucose. Report showed that slow growth of rhizobia associated with woody tree species is related to alkali production, and fast growth is related to acid producers [43]. In the absence of specific taxonomic information, very fast-, fast-, and intermediate-growing (all acid-producing) will be referred to here as *Rhizobium*. In this study, mean generation times of the acid-producing isolate VKLR-01 were within the ranges reported in the literature for *Rhizobium* [38]. 

Isolate VKLR-01 did not grow in media without a carbon source (control). Isolate VKLR-01 utilizes lactose, sucrose, mannitol, D-ribose, maltose, D-galactose, glycerol, sorbitol, sodium citrate, inositol, D-fructose, D-mannose, N-acetyl glucosamine, pyruvate, dextran, *α*-ketoglutarate, and melibiose as the sole carbon sources and L-histidine, L-arginine, and L-proline as the sole nitrogen sources. Isolate VKLR-01 was positive for oxidase, catalase, and nitrate reduction test; however, it showed negative result for gelatin hydrolysis and methyl red tests. The intrinsic antibiotic resistance test for different antibiotics revealed that the isolate VKLR-01 is highly sensitive to antibiotics such as gentamicin, streptomycin, and tetracycline and is resistant to antibiotics like penicillin G, whereas for antibiotics like ampicillin and chloramphenicol it either mildly sensitive or moderately sensitive. Phenotypic characteristics of the isolate is VKLR-01 are shown in Table 1. Isolate VKLR-01 contained ubiquinone-10 (Q-10), at a peak ratio of approximately 100% as the predominant isoprenoid quinone.

### 3.3. FAME Analysis

The most abundant fatty acids are summed feature 8 (65.92%; comprising C_18:1_ *ω*7c and/or C_18:1_ *ω*6c) followed by C_16:0_ iso (10.43%), summed feature 2 (7.42%, comprising C_14:0_ 3OH/C_16:1_ iso I and an unidentified fatty acid with an equivalent chain length of 10.9525) followed by C_16:0_ 3OH (4.19%), C_13:1_ at 12-13 (2.80%), and C_19:0_ cyclo *ω*8c (2.76%), and summed feature 3 (2.42%; comprising C_16:1_ *ω*7c/C_16:1_ *ω*6c). Studies also showed that CFA profile of 5 *Rhizobium* species (*R. soli* DS-42^T^, *R. huautlense* LMG 18254^T^, *R. galegae* LMG 6214^T^, *R. loessense* CIP 108030^T^, and *R. cellulosilyticum* DSM 18291^T^) contains C_18:1_ *ω*7c as the major fatty acid, although there were differences in the proportions of some other fatty acids [9]. The cellular fatty acid profile for isolate VKLR-01 is shown in Table 2.

### 3.4. Genotyping by 16S rRNA, *rec*A, and *atp*D Gene Sequences

**Figure 1:**
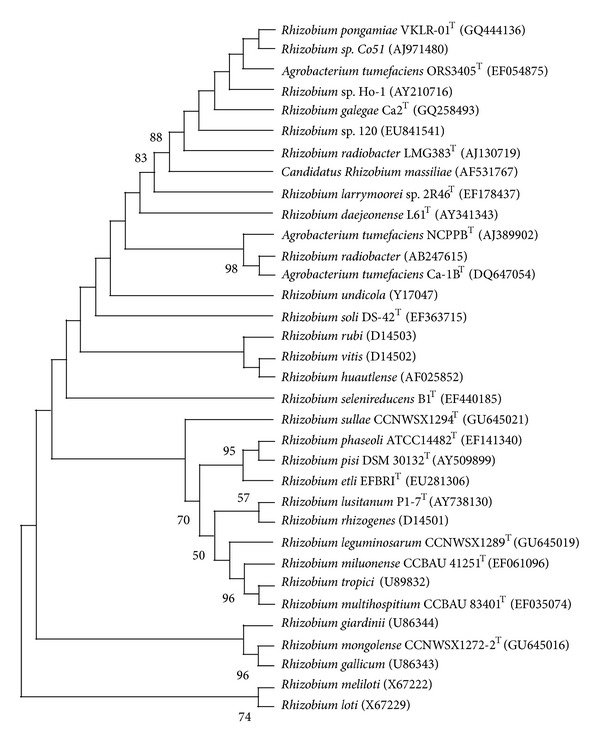
Dendrogram depicting the phylogenetic relationships of *Rhizobium pongamiae* VKLR-01^T^ within the family *Rhizobiaceae* determined using 16S rRNA gene sequence analysis and generated with the MEGA 4.0 software as described in text. Bootstrap values based on 1000 replications are listed as percentages at branching points. Bootstrap values below 50 were omitted from the dendrogram. Bar, 0.002 substitutions per nucleotide position.

Ribosomal RNA is consider the most useful of the highly conserved sequences available for the measurement of phylogenetic relationships [10]. The almost complete 16S rRNA gene sequence of isolate VKLR-01 determined in this study comprised 1428 nucleotides (approximately 95% of the *Escherichia coli* 16S rRNA sequences). In the neighbor-joining tree based on 16S rRNA gene sequences, isolate VKLR-01 fell within the clade comprising *Rhizobium* species (Figure 1). The gene sequence similarities between isolate VKLR-01 and *Rhizobium radiobacter* LMG 383^T^ were 97% and were 94% with *Rhizobium rubi* LMG 156^T^, *Rhizobium alkalisoli *CCBAU 01393^T^, and *Rhizobium vignae* CCBAU 05176^T^, respectively. Gene sequence similarity values of not more than 93% were found when isolate VKLR-01 was compared with other species in the genus *Rhizobium*. The node to which isolate VKLR-01 belonged was also supported in phylogenetic trees generated with the maximum-likelihood and maximum parsimony algorithms (data not shown). In the neighbor-joining tree based on *rec*A gene and *atp*D gene sequences, isolate VKLR-01 formed distinct phylogenetic lineages within the clade comprising *Rhizobium* species (Supplementary Figures 4A and 4B). Isolate VKLR-01 exhibited 81 to 92% *rec*A gene sequence similarity and 80% to 94% *atp*D gene sequence similarity to *Rhizobium* species used in this study, respectively. In the present study, phylogenetic analysis of 16S rRNA housekeeping gene, other housekeeping genes like *atp*D and *rec*A, and other methods of genomic investigations revealed that the isolate VKLR-01 from the root nodules of *P. pinnata* occurring in North Guwahati, Assam, India, represented distinct genotype. These gene sequence similarity values are below the cut-off value of 97%, the level normally judged sufficient to justify the proposal of a novel bacterial species [44]. 

### 3.5. Nitrogen Fixation and Nodulation Assessment Test

In addition to 16S, multilocus sequence analysis (MLSA) is recommended for better resolution of phylogenetic relationships and species identification of novel bacterial strains [45]. In this study, we choose the *nod*D and *nif*H genes and corresponding primers from *R. leguminosarum biovar trifolii, R. leguminosarum biovar viciae,* and *S. meliloti*, which works well with fast-growing rhizobia. Isolate VKLR-01 was able to reduce acetylene to ethylene, and when subjected to *nif*H-specific amplification, it amplified an expected product of 620 bp (Supplementary Figure 5A). The amplified 620 bp fragments of isolate VKLR-01 were sequenced and were found to show 83.0% to 90.0% sequence similarity with other *nif*H sequences from the NCBI database. Nodulation test was performed by PCR amplification of *nod*D gene. Isolate VKLR-01 amplified an expected product of 540 bp (Supplementary Figure 5B). The *nif*H and *nod*D genes amplification results confirm that the isolate VKLR-01 is a nitrogen fixer and plays a role in nodule formation. This will have important implications for biofuel production where reducing inputs (urea-based fertilizers) is highly desirable for production on nutrient-exhausted land [5].

### 3.6. G + C and DNA Hybridization Tests

The DNA G + C content of the isolate VKLR-01 is 59.1 mol% well within the range of values for the genus *Rhizobium* [46]. However, DNA G + C content of the isolate VKLR-01 was lower than that of the type strains *R. soli *DS-42^T^ (60.8 mol%), *R*. *galegae* LMG 6214^T^ (63.0 mol%), *R. loessense* CIP 108030^T^ (59.5 mol%), *R. tianshanense* 6 (63 mol%), and *R. tianshanense* A-1BS^T^ (61 mol%), but higher than that of* R*. *huautlense* LMG 18254^T^ (57 mol%) and *R. cellulosilyticum* DSM 18291^T^ (57 mol%), respectively [9, 47], and it exhibited mean DNA-DNA relatedness values of 51.9% to the type strain of phylogenetically related *Rhizobium* species (*Rhizobium radiobacter* DSM 30147^T^). 

DNA-DNA hybridization provides a useful strategy to establish the taxonomic place and identity of novel strain [48]. Isolate VKLR-01 exhibited mean DNA-DNA relatedness values of 51.9% to the type strain of phylogenetically related *Rhizobium* species (*Rhizobium radiobacter* DSM 30147^T^). Since isolate VKLR-01 shares DNA-DNA hybridization value of less than 70% with reference strain DSM 30147^T^, the isolate is regarded as a distinct *Rhizobium* species [47]. The phylogenetic distinctiveness, together with the DNA-DNA relatedness data and differential phenotypic properties, is sufficient to allocate isolate VKLR-01 to a species that is separate from the recognized *Rhizobium* species and named as *Rhizobium pongamiae *[44]. 

### 3.7. *Rhizobium pongamiae* sp. nov.

On the basis of characterization of phenotypic features, cellular fatty acid profile, cluster analysis, PCR amplification of *nif*H and* nod*D genes, and DNA base composition, DNA-DNA hybridization, the isolate VKLR-01 (= MTCC 10513^T^ = MSCL 1015^T^) from root nodules of *P. pinnata* is considered to represent a novel species within the genus *Rhizobium*. The name for isolate VKLR-01^T^ proposed is *Rhizobium pongamiae* sp. nov. (pon.ga'mi.ae. N.L. gen. n. pongamiae of Pongamia).

## 4. Conclusion

In conclusion, the results based on diverse phenotypic, physiological, biochemical, and molecular studies confirmed the novelty as well as abiotic stress-tolerance potential of the isolated bacterium *Rhizobium pongamiae* (strain VKLR-01^T^) obtained from root nodules of *P. pinnata*, a legume biodiesel crop growing in North Guwahati, Assam, India. Metabolism of the isolate is strictly aerobic and able to fix atmospheric nitrogen and could be defined as a novel species according to the current standards for definition of bacterial (rhizobial) species. The ecological success of the *R. pongamiae* (strain VKLR-01^T^) is that it has specific traits for abiotic stresses, for example, salt, drought, and alkaline tolerance as revealed from the results discussed above which may reflect its advantages for wasteland reclamation, reforestation, and native ecosystem restoration of low-fertility soil. These specific traits of *R. pongamiae* may also be transferred to other rhizobia through biotechnological tools to generate genetic engineered rhizobia beneficial for agricultural point of view.* R. pongamiae* may also be used in several other biotechnological applications such as the production of polysaccharides, enzymes, and antibiotics, which will be the focus of research in future investigations for biotechnological purposes.

## Supplementary Material

Five figures and one Table (Figures S1, S2, S3, S4, & S5; Table S1) in six pages of supplementary material have been included in this file for the paper “*Rhizobium pongamiae* sp. nov. from Root Nodules of *Pongamia pinnata*”. These Figures and Table presents the description of nodule shapes, size, nodule isolate *Rhizobium pongamiae* shape and size by microscope and SEM, its phylogenetic relationships within the family *Rhizobiaceae*, primers sequence information used for amplification and sequencing of 16S rRNA, *rec*A *atp*D, *nif*H and *nod*D genes from *Rhizobium pongamiae*.

## Figures and Tables

**Table 1:** Phenotypic characteristics of *Rhizobium pongamiae* VKLR-01^T^ and type strains of phylogenetically related *Rhizobium* species.

Characteristic	1	2	3	4
Origin	Root nodule of *P. pinnata* (North Guwahati, Assam)	*Galega orientalis* (Finland)	ND	*Ficus benjamina *(Florida)
Cell morphology (*µ*m)	Rods (0.4–0.5 × 1.4–1.6)	Rods (0.9–1.0 × 1.5–1.8)^a^	Rods (0.6–1 × 1.5–3.0)	Rods
Flagella	ND	1-2	Several, peritrichous	Several, peritrichous
Nodulation	+	+	−	−
pH range	6–11	5.0–9.5^a^	ND	ND
Growth at/in				
40°C	+	−	ND	−
1% NaCl	+	+	+	+
2% NaCl	+	−	+	+
4% NaCl	+	−	−	−
Utilization as carbon source				
Sucrose	+	+	ND	ND
Arabinose	−	+	+	ND
Mannitol	+	+	+	ND
Lactose	+	−	ND	ND
Glucose	−	+	ND	ND
Maltose	+	ND	+	+
Melibiose	+	+	+	ND

Strains: 1, *R. pongamiae *VKLR-01^T^ (present study); 2, *R. galegae *LMG 6214^T^ 3; 3, *A. tumefaciens *(biovar 1)*; *4, *R. larrymoorei *AF3-10^T^ [9–12]. ^a^Unpublished data of Wang et al. (personal communication) [13]. +: positive; −: negative; ND: data not available. All strains are Gram negative, aerobic, rod shaped, and nonspore forming. All 7 strains are positive for oxidase, catalase, and nitrate reductase tests.

**Table 2:** Cellular fatty acid composition (%) of *Rhizobium pongamiae* VKLR-01^T^ and type strains of phylogenetically related *Rhizobium* species.

Fatty acid	1	2	3
Straight-chain fatty acid			
12:0	00.55	—	—
14:0	00.75	—	0.11
15:0	—	—	0.29
16:0	10.43	7.7	9.03
17:0			0.13
18:0	00.66	0.9	0.17
Unsaturated fatty acid			
13:1 at 12-13	02.80	—	0.49
16:1 *ω*5c	—	—	—
17:1 *ω*8c	00.52	—	—
15:1 *ω*8c	—	0.8	—
17:1 *ω*6c	—	—	—
17:1 *ω*8c	—	—	0.22
18:1 *ω*5c	—	—	—
18:1 *ω*7c	—	76.2	—
Hydroxy fatty acid	—	—	—
12:0 3-OH	—	—	—
13:0 2-OH	—	—	—
15:0 3-OH	—	—	0.03
15:1 3-OH iso	—	—	—
16:0 3-OH	04.19	2.0	4.76
17:0 3-OH	—	—	0.18
18:0 2-OH	—	—	—
18:0 3-OH	00.58	0.7	—
15:1 G iso	—	—	—
16:0 iso	00.67	—	—
10-Methyl 18:0 TBSA	00.34	—	—
10-Methyl 19:0	—	2.1	1.09
11-Methyl 18:1 *ω*7c	—	—	0.23
17:0 cyclo	—	—	1.60
19:0 cyclo *ω*8c	02.76	3.9	18.78
20:2 *ω*6, 9c	—	—	0.01
20:3 *ω*6, 9, 12c	—	—	0.33
Unknown (ECL 18.794)	—	—	—
Summed features*	—	—	—
2	7.41	4.6	8.18
3	02.42	0.52	—
4	—	—	1.62
7	—	—	52.41
8	65.92	—	—

Strains: 1, *R. pongamiae *sp. nov.; 2, *R. galegae *LMG 6214^T^ 12 [9]; 3, *A. tumefaciens *[14]. Values are percentages of the total amount of fatty acid compounds present for those 14 species. ECL: equivalent chain length.

—: not detected.

*Summed features 2 (12:0 aldehyde? and/or 16:1 iso I and/or 14:0 3-OH and/or unknown ECL 10.928 and/or unknown ECL 10.9525 and/or 15:1 iso H/I, 13:0 3-OH).

*Summed features 3 (16:1 *ω*7c and/or 16:1 *ω*6c and/or 15:0 iso 2-OH).

*Summed features 4 (iso-17:1 I and/or anteiso-17:1 B and/or 15:0 iso 2-OH, 16:1 *ω*7c).

*Summed features 7 (18:1 *ω*7c and/or *ω*9 trans and/or *ω*12 trans and/or 18:1 *ω*7c and/or *ω*9c and/or *ω*12trans).

*Summed features 8 (18:1 *ω*7c and/or 18:1 *ω*6c).
