# Supplementary material for: Rhizobium pongamiae sp. nov. from Root Nodules of Pongamia pinnata
Source: Biomed Res Int. 2013 Jul 2;2013:165198. doi: 10.1155/2013/165198 (PMC3783817; doi:10.1155/2013/165198)
Supplement: Supplementary file 1 — Five figures and one Table (Figures S1, S2, S3, S4, & S5; Table S1) in six pages of supplementary material have been included in this file for the paper “Rhizobium pongamiae sp. nov. from Root Nodules of Pongamia pinnata”. These Figures and Table presents the description of nodule shapes, size, nodule isolate Rhizobium pongamiae shape and size by microscope and SEM, its phylogenetic relationships within the family Rhizobiaceae, primers sequence information used for amplification and sequencing of 16S rRNA, recA atpD, nifH and nodD genes from Rhizobium pongamiae. [file 165198.f1.pdf]

Supplementary Table 1. Primers sequence information used for amplification and sequencing of 16S rRNA, *rec*A, *atp*D, *nif*H and *nod*D genes.

Gene name	Primer sequence (5'->3')	
	**Forward**	**Reverse**
**16S rDNA**	TGAGACACGACCCACTCTAC	AAGAGCTGGTAAGGTTCTGC
***rec*A**	ATCGAGCGGTCGTTCGGCAAGGG	TTGCGCAGCGCCTGGCTCAT
***atp*D**	ATCGGCGAGCCGGTCGACGA	GCCGACACTTCCGAACCAGCCTG
***nif*H**	AAGACGTGCTCAAGGTCGGC	GGGATGGTGCCTTTGCCAGA
***Nod*D**	ATCAACCTCAGCCAACCCGC	CACGTCCGAACTTAGCCGCA
