# Supplementary material for: Rhizobium pongamiae sp. nov. from Root Nodules of Pongamia pinnata
Source: Biomed Res Int. 2013 Jul 2;2013:165198. doi: 10.1155/2013/165198 (PMC3783817; doi:10.1155/2013/165198)
Supplement: Supplementary file 5 [file 165198.f5.ppt]

## Slide 1
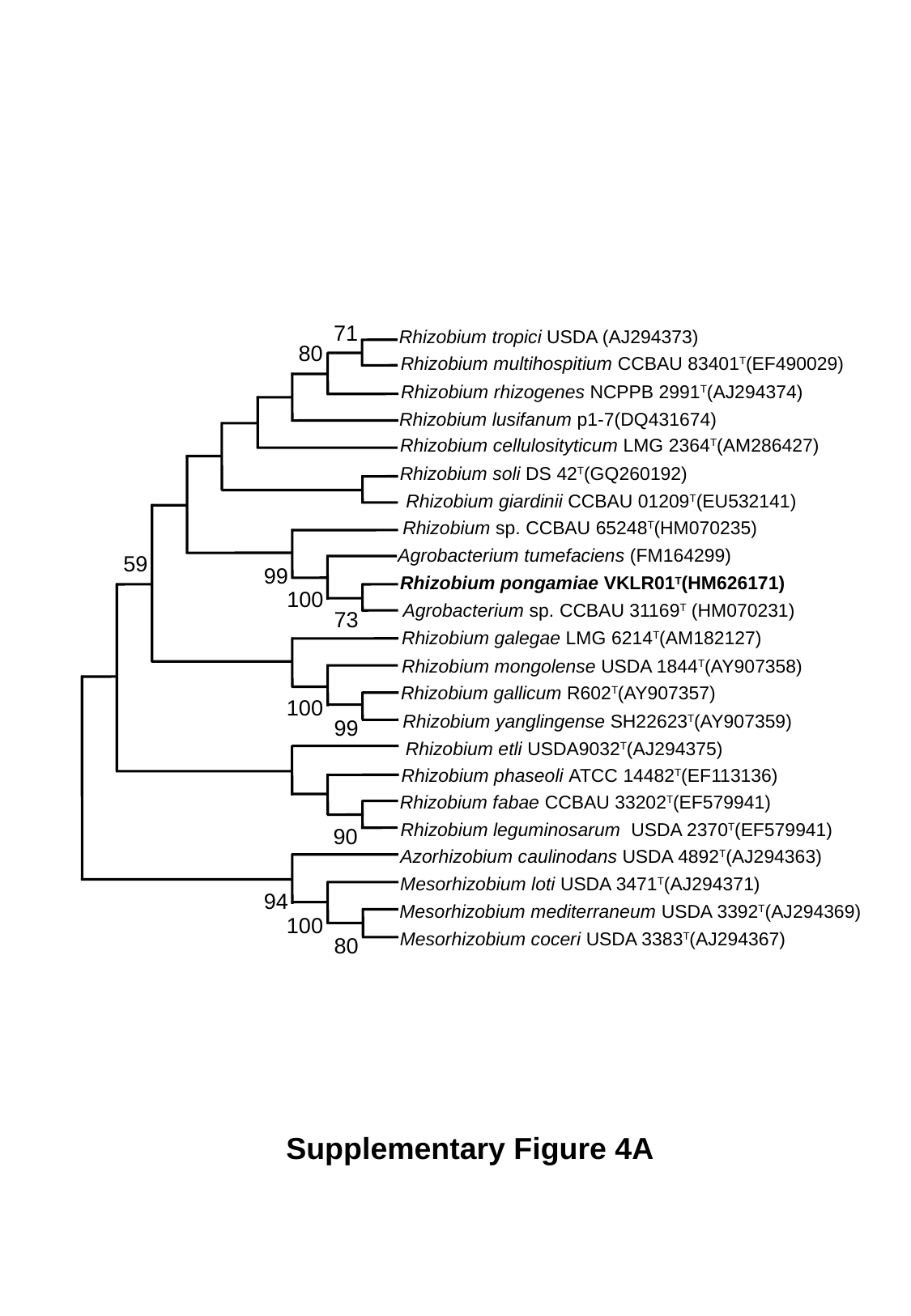

71
Rhizobium tropici USDA (AJ294373)
80
Rhizobium multihospitium CCBAU 83401T(EF490029)
Rhizobium rhizogenes NCPPB 2991T(AJ294374)
Rhizobium lusifanum p1-7(DQ431674)
Rhizobium cellulosityticum LMG 2364T(AM286427)
Rhizobium soli DS 42T(GQ260192)
Rhizobium giardinii CCBAU 01209T(EU532141)
Rhizobium sp. CCBAU 65248T(HM070235)
Agrobacterium tumefaciens (FM164299)
59
99
Rhizobium pongamiae VKLR01T(HM626171)
100
Agrobacterium sp. CCBAU 31169T (HM070231)
73
Rhizobium galegae LMG 6214T(AM182127)
Rhizobium mongolense USDA 1844T(AY907358)
Rhizobium gallicum R602T(AY907357)
100
Rhizobium yanglingense SH22623T(AY907359)
99
Rhizobium etli USDA9032T(AJ294375)
Rhizobium phaseoli ATCC 14482T(EF113136)
Rhizobium fabae CCBAU 33202T(EF579941)
Rhizobium leguminosarum USDA 2370T(EF579941)
90
Azorhizobium caulinodans USDA 4892T(AJ294363)
Mesorhizobium loti USDA 3471T(AJ294371)
94
Mesorhizobium mediterraneum USDA 3392T(AJ294369)
100
Mesorhizobium coceri USDA 3383T(AJ294367)
80
Supplementary Figure 4A
