# Supplementary material for: Rhizobium pongamiae sp. nov. from Root Nodules of Pongamia pinnata
Source: Biomed Res Int. 2013 Jul 2;2013:165198. doi: 10.1155/2013/165198 (PMC3783817; doi:10.1155/2013/165198)
Supplement: Supplementary file 6 [file 165198.f6.ppt]

## Slide 1
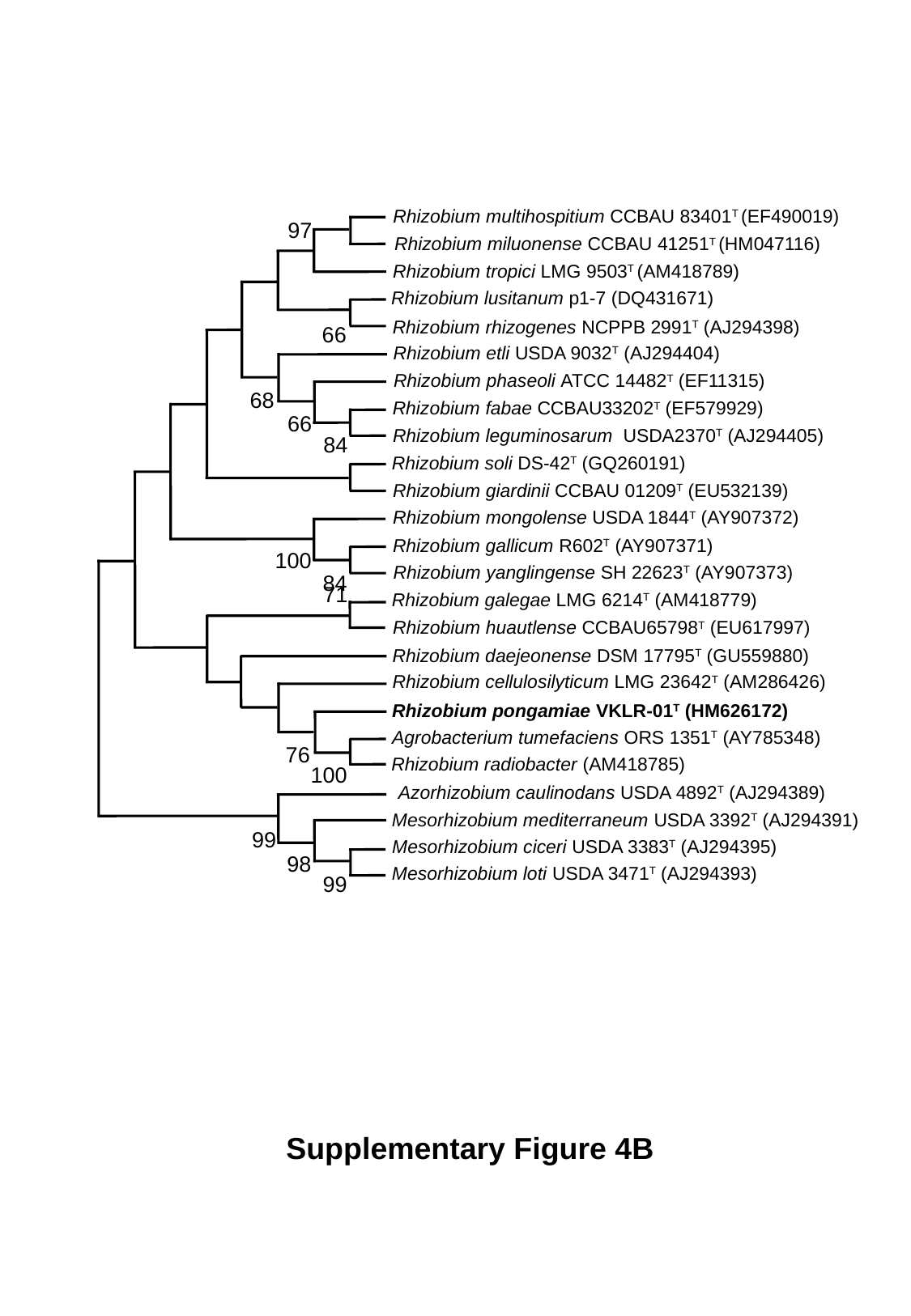

Rhizobium multihospitium CCBAU 83401T (EF490019)
97
Rhizobium miluonense CCBAU 41251T (HM047116)
Rhizobium tropici LMG 9503T (AM418789)
Rhizobium lusitanum p1-7 (DQ431671)
Rhizobium rhizogenes NCPPB 2991T (AJ294398)
66
Rhizobium etli USDA 9032T (AJ294404)
Rhizobium phaseoli ATCC 14482T (EF11315)
68
Rhizobium fabae CCBAU33202T (EF579929)
66
Rhizobium leguminosarum USDA2370T (AJ294405)
84
Rhizobium soli DS-42T (GQ260191)
Rhizobium giardinii CCBAU 01209T (EU532139)
Rhizobium mongolense USDA 1844T (AY907372)
Rhizobium gallicum R602T (AY907371)
100
Rhizobium yanglingense SH 22623T (AY907373)
84
71
Rhizobium galegae LMG 6214T (AM418779)
Rhizobium huautlense CCBAU65798T (EU617997)
Rhizobium daejeonense DSM 17795T (GU559880)
Rhizobium cellulosilyticum LMG 23642T (AM286426)
Rhizobium pongamiae VKLR-01T (HM626172)
Agrobacterium tumefaciens ORS 1351T (AY785348)
76
Rhizobium radiobacter (AM418785)
100
Azorhizobium caulinodans USDA 4892T (AJ294389)
Mesorhizobium mediterraneum USDA 3392T (AJ294391)
99
Mesorhizobium ciceri USDA 3383T (AJ294395)
98
Mesorhizobium loti USDA 3471T (AJ294393)
99
Supplementary Figure 4B
