# Supplementary figures and images for: Rhizobium pongamiae sp. nov. from Root Nodules of Pongamia pinnata
Source: Biomed Res Int. 2013 Jul 2;2013:165198. doi: 10.1155/2013/165198 (PMC3783817; doi:10.1155/2013/165198)

## Slide 1
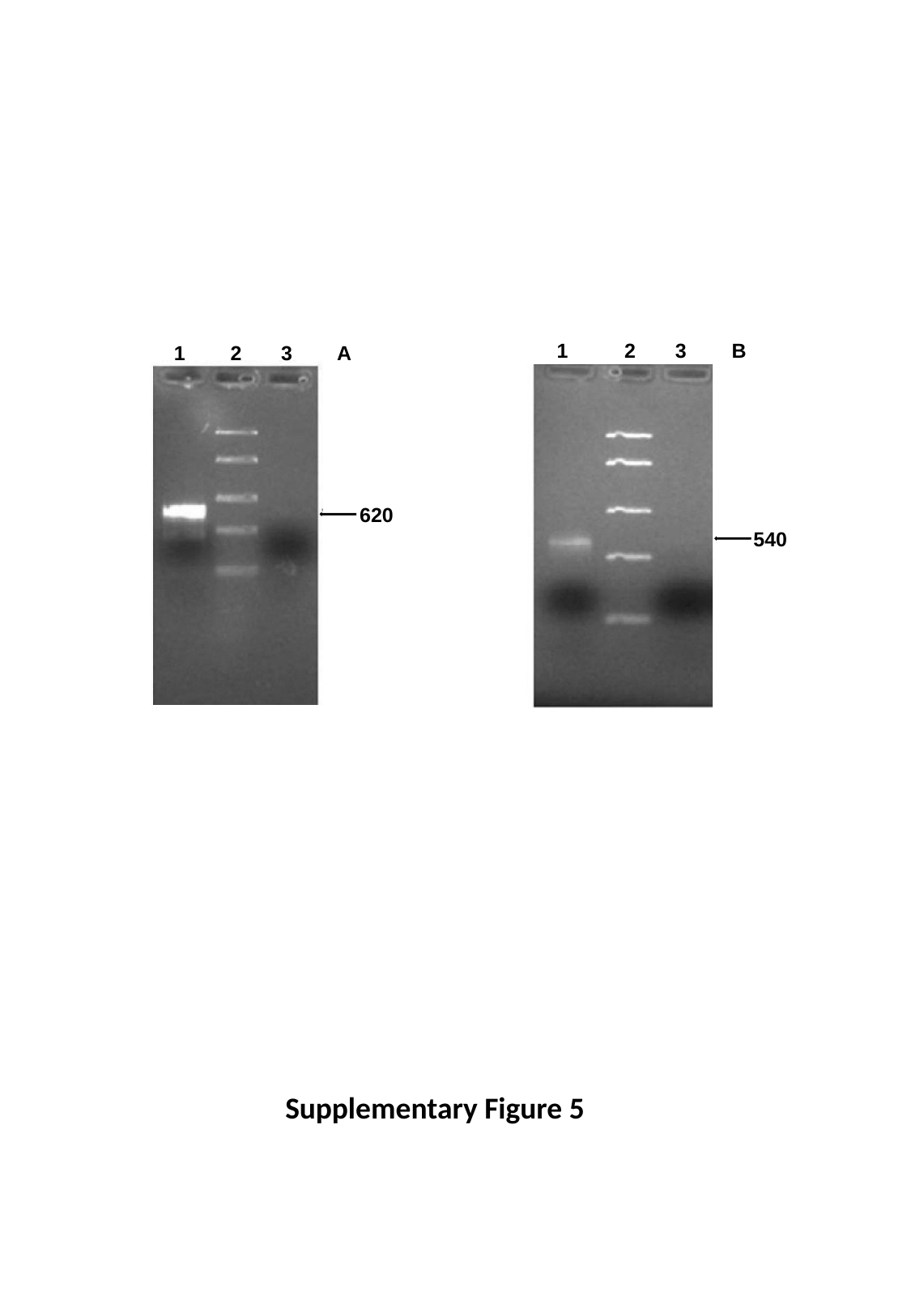

1 2 3 B
1 2 3 A
620
540
Supplementary Figure 5

Supplement: Supplementary file 7 [file 165198.f7.ppt]
